# Serum and urinary essential trace elements in association with major depressive disorders: a case–control study

**DOI:** 10.3389/fpsyt.2023.1297411

**Published:** 2023-12-01

**Authors:** Jiyong Fu, Qinqin Wang, Na Wang, Shilong Li, Hongwei Zhang, Yuxing Zhu, Hua Guo, Fukun Wang, Lei He, Shuang Xia, Bing Cao

**Affiliations:** ^1^Zhumadian Second People's Hospital, Zhumadian, Henan, China; ^2^Key Laboratory of Cognition and Personality, Faculty of Psychology, Ministry of Education, Southwest University, Chongqing, China

**Keywords:** major depressive disorder, essential trace elements, serum, urine, case–control

## Abstract

**Introduction:**

The etiology and pathophysiology of major depressive disorders (MDDs) remain unclear. Increasing evidence has demonstrated that essential trace elements (ETEs), such as iodine (I), zinc (Zn), copper (Cu), selenium (Se), cobalt (Co), and molybdenum (Mo), play vital roles in MDDs.

**Methods:**

In total, 72 patients with MDD and 75 healthy controls (HCs) in the Zhumadian Second People's Hospital, Henan Province, China were recruited in our study. The levels of different ETEs were examined in both serum and urine, using an inductively coupled plasma mass spectrometer (ICP-MS), for both the MDD patients and HCs.

**Results:**

The serum levels of I, Se, Cu, and Mo were significantly lower in the MDD patients compared to the HCs (*p* < 0.05), and the urinary levels of I and Zn were significantly higher in the MDD patients compared to the HCs (*p* < 0.05). The serum concentration of I (Q3: OR = 0.210, Q4: OR = 0.272) was negatively associated with MDD after adjusting for potential confounders, including age, gender, and BMI, and the urinary concentration of I (Q4: OR = 2.952) was positively associated.

**Conclusions:**

The higher levels of I, Se, Cu, and Mo in serum might be protective against the development of MDD, and the excess I and Zn in urine may be associated with MDD pathogenesis. Future research needs to gain a deeper understanding of the metabolic pathways of ETEs, especially I, Se, Zn, Cu, and Mo, in MDD, and their role in the pathogenesis of depression.

## 1 Introduction

Depression is usually defined as a condition characterized by low mood, decreased energy levels, and reduced exercise and physical activity with impaired motivation, reward, and arousal. A Major depressive disorder (MDD) can be defined as a highly complicated disease that affects the entire life of a patient, such as mood, behavior, cognition, sleep, and other physical functions (1). The WHO predicts that depression will top the global burden of disease by 2030. Currently, the main causes of depression are still unclear and need extensive research and comprehension.

Essential trace elements (ETEs) are present in concentrations below 1000 ppm that originate from natural anthropogenic activities, are transported to areas through atmospheric and geochemical processes, and are made bioavailable to organisms through uptake at the base trophic levels (2–4). These elements, which include dietary minerals, are required in very minute quantities for proper growth, development, and physiology of organisms (5). The trace elements play crucial catalytic and structural roles. Additionally, they may act as cofactors for most of the enzymatic reactions in the body. For example, zinc (Zn) is necessary for the metabolism of proteins and carbohydrates (6). Previous studies reported that Zn and Cu have the potential to modulate glutamate receptors and transporters (7, 8), especially the glutamatergic N-methy1-D-aspartate (NMDA) receptor, which was inhibited by Zn and Cu ions (9, 10). The disturbance of glutamatergic transmission via NMDA receptor may be the pathogenesis of mood disorders (8). Furthermore, depressive symptoms caused by deficiency in Zn have also been reported previously (11). For example, the inflammatory response as a symptom of depression has been linked to lower Zn concentration in serum (12), and the Cu ions involved in the inflammatory process can play a role as well (13). ETEs such as Cu, Zn, and Fe are essential for the growth and development of the human body (14). These elements are crucial for various physiological process and play a major role in the functioning of cells. Deficiency or abundance of these elements may result in a variety of diseases including depression (15). In the bipolar disorders, changes in trace elements also play important roles, with high Cu/Zn ratios commonly observed (16).

Several studies have shown that the concentrations of ETEs in serum levels are correlated with the etiology and pathophysiology of major depression (17, 18). For example, iodine (I) is an essential micronutrient and an integral building block of the thyroid hormones, which regulate multiple metabolic processes that are important for growth, metabolism, and reproduction, and insufficient maternal habitual I intake has been associated with symptoms of perinatal depression (19); an increased risk of depressive symptoms can be associated with elevated selenium (Se) levels in serum (20), and interestingly, a U-shaped relationship between serum levels of Se and depressive symptoms has also been observed (21); lower serum Zn levels in depression cases may lead to neuronal deficits, and abnormal Zn levels may take a potential role in the pathophysiology of depression (22). Previous studies suggested that Zn, Fe, and Se deficiencies are the main cause of oxidative stress and, in the long run, manifest themselves in the development of bipolar disorders (23). Although the relationships between ETEs and MDD in serum have been extensively studied, their associations in urine are rarely explored. Only few studies reported the correlation between ETEs and depression in urine. For example, high urinary I concentration may be linked to an increased risk of major depression among older adults (24).

Increasing evidence demonstrated that some ETE changes can be associated with MDD, but most studies focused on samples of serum, hair, or nails, studies on the levels of ETEs in the urine of depressed patients are limited. The current study aims to compare the differences between different ETEs, including I, Fe, Zn, Se, Cu, Mo, and Co, in urine and serum of MDD patients. Additionally, the differences between different ETE levels in MDD patients and HCs were examined. The results of this study may provide some referable recommendations for the treatment and prevention of MDD.

## 2 Methods

### 2.1 Ethical approval

The study protocols have been reviewed and approved by the Medical Ethics Committee of Zhumadian Second People's Hospital in Henan Province (approval no. IRB-2021-006-02). The MDD patients and controls were provided with an informed written consent form before they participated in the study. Additionally, all procedures adhered to the standards set forth by the Helsinki Declaration.

### 2.2 Participants

Our study involved a total of 147 individuals, including 72 patients (36 men and 36 women) diagnosed with MDD and 75 healthy controls in the Zhumadian Second People's Hospital in Henan Province. The study had the following criteria for participant inclusion: (1) adherence to the Diagnostic and Statistical Manual of Mental Disorders, 5th edition (DSM-5); (2) first episode or relapse without taking antidepressants or antipsychotics within a month; (3) education level above primary school; (4) Hamilton Depression Scale (HAMD) - 24 version score ≥ 20; (5) age: 18–60 years old, with no gender limitation. Healthy controls (49 women, 26 men), met the following inclusion criteria: (1) matching the gender, age, and place of residence of the MDD patient group; (2) primary school education or above; (3) routine hematology, urine, and feces, liver function, fasting blood glucose, renal function, chest x-ray, electrocardiogram, etc. The exclusion criteria are the same as for the MDD group.

The exclusion criteria of all participants are as follows: (1) history of organic brain disease or diagnosed neurologic disorder (e.g., Parkinson's, cerebral hemorrhage, massive cerebral infarction, encephalitis, or epilepsy); (2) severe medical conditions that are clinically significant or unstable, including liver, kidney, gastrointestinal, respiratory, cardiovascular, endocrine, blood, neurological, genitourinary, musculoskeletal, or metabolic-related diseases and problems; (3) intellectual disabilities; (4) history of alcohol, drugs, chemicals, substances, or psychoactive substance abuse; (5) vision or hearing impairment; and (6) pregnant and lactating women.

### 2.3 Basic and clinical information collection

Trained healthcare workers collected general information about all participants, including their gender, age, body mass index, occupation, and marital status; they also collected information about the participants' family history of mental disorders, parental literacy, and parental marital status; and basic information about the participants' infancy and childhood was also gathered. The HAMD-24 was used to measure the severity of psychiatric symptoms, and clinical information, such as routine blood, blood biochemistry, and urine tests, was collected from all participants.

### 2.4 Sample and detection

Essential trace element levels of I, Fe, Zn, Se, Cu, Mo, and Co in the serum and urine of individuals were measured. All participants provided 8.5 ml of intravenous morning blood once in the morning (7–9 a.m.) after 12 h fasting for serum isolation. Additionally, 10 ml of morning urine was collected using a sterile catheter. The serum was collected intravenously using golden yellow inert separation gel collection blood vessels. After coagulation at 4°C for one h, the upper serum samples were separated by centrifugation at 4°C for 10 min. They were then aliquoted in 5 ml cryopreservation tubes and stored in a −80°C refrigerator for the measurement of trace elements.

The operation of the experiment is as follows: A 0.1 ml serum (or urinary) sample is taken into a 2 ml centrifuge tube. Then, 0.1 ml of a combined internal standard of indium (In), rhenium (Re) is added, along with 1% nitric acid. The mixture is shaken well and measured on the machine. We used Perkin-Elmer Sciex's Elan DRC II inductively coupled plasma mass spectrometer (ICP-MS) and Agilent's 7700 × ICP-MS to analyze essential trace elements.

### 2.5 Statistical analysis

The statistical analysis was conducted using SPSS 28.0 (Statistical Package for Social Sciences). All analyses were considered with a 95% confidence interval (CI), and the significance level was double-tailed *p* < 0.05. In the evaluation of the data, continuous variables were summarized as the mean and standard deviation (SD) or median and interquartile range (IQR), and categorical variables were summarized as frequencies and proportions (*N*, %). We conducted a normality test on the data before performing inferential statistics. The independent samples *t*-test was used to compare the differences between two groups for normally distributed continuous variables, and the Mann–Whitney *U-*test was used to compare the differences between two groups for no-normally distributed continuous variables. A chi-squared test was used between two groups by giving the frequency distribution of categorical data. The median with lower and upper quartiles was described as the concentrations of all ETEs, for the concentrations of the elements were not normally distributed. Age, gender, and body mass index were included in the analysis as covariates. Spearman's rank correlations analysis also was used to examine correlations between different elements.

The HCs were categorized into four groups by using the quartiles of element concentrations, and then according to the concentrations of elements, the MDD patients were categorized into four different groups. We used the Q1 group as a reference, and the other groups performed logistics regression separately from the Q1 group. The concentrations of different trace elements in serum or urine can be analyzed in this manner.

## 3 Results

### 3.1 Demographic and clinical characteristics of MDD patients and HCs

In total, 72 MDD cases and 75 healthy controls were included in the analysis. The mean ages (SD) of MDD patients and healthy controls were 39.30 ± 15.50 years and 41.90 ± 6.90 years, respectively. The HAMD-24 (MDD cases: 26.80 ±6.0; HCs: 2.10 ±1.50; *p* = 0.00) and BMI (cases: 22.20 ± 4.30 kg/m^2^; HCs: 23.90 ± 2.10 kg/m^2^; *p* = 0.00) values of the two groups were statistically significant (Table 1); however, the distribution of age and sex were not statistically significant among participants (all *p* > 0.05).

**Table 1:** Demographic characteristics of participants.

**Variable**	**Healthy controls (*n =* 75)**	**Depression (*n =* 72)**	***p*-values**
Age, year, Mean ± SD	41.9 ± 6.9	39.3 ± 15.5	0.178
Sex (female/male); *n*/%	49/26 (65.3/34.7)	36/36 (50.0/50.0)	0.060
HAMD-24 total score, Mean ± SD	2.1 ±1.5	26.8 ±6.0	< 0.001
BMI, kg/m^2^, Mean ± SD	23.9 ± 2.1	22.2 ± 4.3	0.004

### 3.2 The concentrations of ETEs in serum of the MDD cases and HCs

The concentrations of I (MDD patients: 65.96 ng/mL, IQR: 58.57–73.72 ng/mL; HCs: 75.80 ng/mL, IQR: 68.32–83.31 ng/mL; *p* = 0.00), Se (MDD patients: 113.05 ng/mL, IQR: 100.10–130.09 ng/mL; HCs: 125.72 ng/mL, IQR: 106.19–145.96 ng/mL; *p* = 0.02), Cu (MDD patients: 992.16 ng/mL, IQR: 843.61–1170.65 ng/mL; HCs: 1271.60 ng/mL, 1151.04–1432.42 ng/mL; *p* = 0.00), and Mo (MDD patients: 1.22 ng/mL, 1.03–1.54 ng/mL; HCs: 1.43 ng/mL, 1.12–1.72 ng/mL; *p* = 0.01) were found to be lower in MDD patients compared to the HCs. The concentrations of other ETEs, such as Fe, Zn, and Co, in these two groups were no statistically significant (*p* > 0.05) (Table 2).

**Table 2:** The concentrations of ETEs between the MDD cases and HCs in serum or urine.

**ETEs**	**Serum**			**Urinary**		
	**Healthy controls (*****n** =* **75)**	**Depression (*****n** =* **72)**	***p-*** **values**	**Healthy controls (*****n** =* **75)**	**Depression (*****n** =* **72)**	***p-*** **values**
I (ng/mL, IQR)	75.80 (68.32, 83.31)	65.96 (58.57, 73.72)	**< 0.001**	191.92 (127.54, 275.67)	221.14 (139.49, 353.88)	**0.038**
Fe (ng/mL, IQR)	1319.90 (1105.06, 1660.95)	1316.01 (948.85, 1562.19)	0.122	348.47 (265.57, 468.62)	373.04 (261.59, 564.79)	0.229
Zn (ng/mL, IQR)	1080.10 (925.68, 1338.41)	1116.73 (965.89, 1289.14)	0.535	316.77 (177.48, 573.61)	477.18 (259.59, 1068.24)	**0.015**
Se (ng/mL, IQR)	125.72 (106.19, 145.98)	113.05 (100.10, 130.09)	**0.020**	23.29 (16.81, 29.61)	23.98 (15.46, 34.74)	0.509
Cu (ng/mL, IQR)	1271.60 (1151.04, 1432.42)	992.16 (843.610, 1170.65)	**< 0.001**	34.63 (23.33, 45.74)	29.38 (20.21, 40.56)	0.077
Mo (ng/mL, IQR)	1.43 (1.12, 1.72)	1.22 (1.03, 1.54)	**0.014**	33.46 (21.42, 52.41)	40.95 (23.62, 58.11)	0.188
Co (ng/mL, IQR)	0.56 (0.46, 0.66)	0.51 (0.43, 0.63)	0.058	0.43 (0.26, 0.83)	0.43 (0.22, 0.69)	0.564

IQR, inter-quartile range, unit: ng/mL serum. Bold represents *p* < 0.05.

### 3.3 The urinary concentrations of ETEs in the MDD patients and HCs

The level of I in urine was significantly higher in MDD patients (221.14 ng/mL, 139.49–353.88 ng/mL) compared to HCs (191.92 ng/mL, 127.54–275.67 ng/mL) (*p* < 0.04), which contrasts with previous serum findings. The concentration of Zn (MDD patients: 477.18 ng/mL, 259.59–1068.24 ng/mL; HCs: 316.77 ng/mL, 177.48–573.61 ng/mL; *p* = 0.02) in urine was significantly higher in MDD cases compared to the HCs. However, there were no statistically significant differences in the concentrations of Fe, Se, Cu, Mn, and Co (*p* > 0.05) between MDD patients and controls (Table 2).

### 3.4 The association between serum and urinary concentrations of ETEs and MDD

The results of logistic regression analysis revealed that, after adjusting for age, gender, and BMI, the OR of I (Q3: OR = 0.21, 95% CI: 0.08, 0.58, *p* = 0.00; Q4: OR = 0.27, 95% CI: 0.11, 0.70, *p* = 0.01) in serum was < 1.0 in MDD patients compared to HCs, while in urine, the OR of I (Q4: OR = 2.95, 95% CI: 1.23, 7.07, *p* = 0.02) in MDD patients was >1.0. The OR of Zn (OR = 2.95, 95% CI: 1.14, 7.63, *p* = 0.03) in urine was >1.00, but not significantly different in serum. The analysis indicated that the ORs of Cu in the serum were lower than 1.00 (Q2: OR = 0.10, 95% CI: 0.03, 0.32, *p* = 0.00; Q3: OR = 0.04, 95% CI: 0.01, 0.20, *p* = 0.00; Q4: OR = 0.24, 95% CI: 0.10, 0.60, *p* = 0.00) for MDD patients compared to HCs. However, there was no significant difference in the urine. All data has been adjusted for covariates such as sex, age, and BMI (Table 3).

**Table 3:** Logistic regression analysis of serum and urinary concentrations of ETEs in MDD patients and HCs.

**ETEs**		**Serum**				**Urinary**			
		**OR** ^a^	***p***	**AOR**	***p***	**OR** ^b^	***p***	**AOR**	***p***
I	Q1	1.000		1.000		1.000		1.000	
	Q2	0.842 (0.433, 1.638)	0.613	0.506 (0.215, 1.188)	0.118	0.667 (0.321, 1.384)	0.277	1.042 (0.394, 2.756)	0.934
	Q3	0.368 (0.155, 0.876)	**0.024**	0.210 (0.076, 0.579)	**0.003**	0.55 (0.264, 1.148)	0.111	0.839 (0.315, 2.235)	0.726
	Q4	0.474 (0.214, 1.047)	0.065	0.272 (0.106, 0.701)	**0.007**	1.833 (1.032, 3.256)	**0.039**	2.952 (1.233, 7.071)	**0.015**
Fe	Q1	1.000		1.000		1.000		1.000	
	Q2	0.556 (0.256, 1.203)	0.136	0.494 (0.186, 1.31)	0.157	0.526 (0.245, 1.132)	0.100	0.634 (0.237, 1.697)	0.364
	Q3	1.1 (0.6, 2.015)	0.758	0.98 (0.428, 2.246)	0.962	1 (0.529, 1.889)	1.00	1.208 (0.503, 2.898)	0.673
	Q4	0.778 (0.387, 1.564)	0.481	0.686 (0.278, 1.694)	0.414	1.211 (0.659, 2.223)	0.538	1.461 (0.606, 3.521)	0.398
Zn	Q1	1.000		1.000		1.000		1.000	
	Q2	1 (0.529, 1.889)	1	1.881 (0.732, 4.833)	0.19	0.632 (0.307, 1.301)	0.213	1.328 (0.471, 3.741)	0.592
	Q3	1.421 (0.79, 2.556)	0.241	2.678 (1.107, 6.481)	**0.029**	1.105 (0.594, 2.056)	0.752	2.37 (0.895, 6.274)	0.082
	Q4	0.722 (0.354, 1.474)	0.371	1.233 (0.466, 3.264)	0.673	1.421 (0.79, 2.556)	0.241	2.951 (1.141, 7.63)	**0.026**
Se	Q1	1.000		1.000		1.000		1.000	
	Q2	1.55 (0.883, 2.719)	0.127	1.925 (0.832, 4.453)	0.126	0.737 (0.369, 1.47)	0.386	0.871 (0.342, 2.22)	0.772
	Q3	0.684 (0.338, 1.385)	0.292	0.866 (0.334, 2.247)	0.768	0.579 (0.276, 1.217)	0.149	0.713 (0.268, 1.898)	0.499
	Q4	0.5 (0.225, 1.113)	0.09	0.585 (0.207, 1.649)	0.31	1.421 (0.79, 2.556)	0.241	1.761 (0.728, 4.259)	0.209
Cu	Q1	1.000		1.000		1.000		1.000	
	Q2	0.278 (0.103, 0.748)	**0.011**	0.101 (0.032, 0.322)	**< 0.001**	1.053 (0.562, 1.972)	0.873	1.062 (0.444, 2.539)	0.892
	Q3	0.105 (0.025, 0.452)	**0.002**	0.041 (0.009, 0.195)	**< 0.001**	0.789 (0.401, 1.554)	0.494	0.809 (0.32, 2.046)	0.654
	Q4	0.632 (0.307, 1.301)	0.213	0.24 (0.096, 0.596)	**0.002**	0.722 (0.354, 1.474)	0.371	0.723 (0.28, 1.863)	0.502
Mo	Q1	1.000		1.000		1.000		1.000	
	Q2	1.5 (0.826, 2.723)	0.183	1.507 (0.647, 3.509)	0.341	0.789 (0.401, 1.554)	0.494	1.336 (0.515, 3.466)	0.551
	Q3	0.7 (0.354, 1.386)	0.306	0.68 (0.269, 1.719)	0.415	1.167 (0.622, 2.19)	0.631	2.024 (0.799, 5.129)	0.137
	Q4	0.389 (0.162, 0.931)	**0.034**	0.384 (0.133, 1.107)	0.076	1.105 (0.594, 2.056)	0.752	1.875 (0.744, 4.727)	0.182
Co	Q1	1.000		1.000		1.000		1.000	
	Q2	1.421 (0.79, 2.556)	0.241	1.503 (0.654, 3.451)	0.337	0.833 (0.42, 1.653)	0.602	1.055 (0.417, 2.671)	0.91
	Q3	0.368 (0.155, 0.876)	0.024	0.389 (0.135, 1.116)	0.079	1.316 (0.725, 2.389)	0.367	1.639 (0.7, 3.839)	0.255
	Q4	0.842 (0.433, 1.638)	0.613	0.923 (0.378, 2.255)	0.86	0.632 (0.307, 1.301)	0.213	0.809 (0.305, 2.15)	0.671

^a^Determined by an unconditional logistic regression model. ^b^Adjusted OR and 95% CI were determined by an unconditional logistic regression model after adjusting for age, gender, and BMI. Bold represents *p* < 0.05.

### 3.5 Correlation analysis

**Figure 1:**
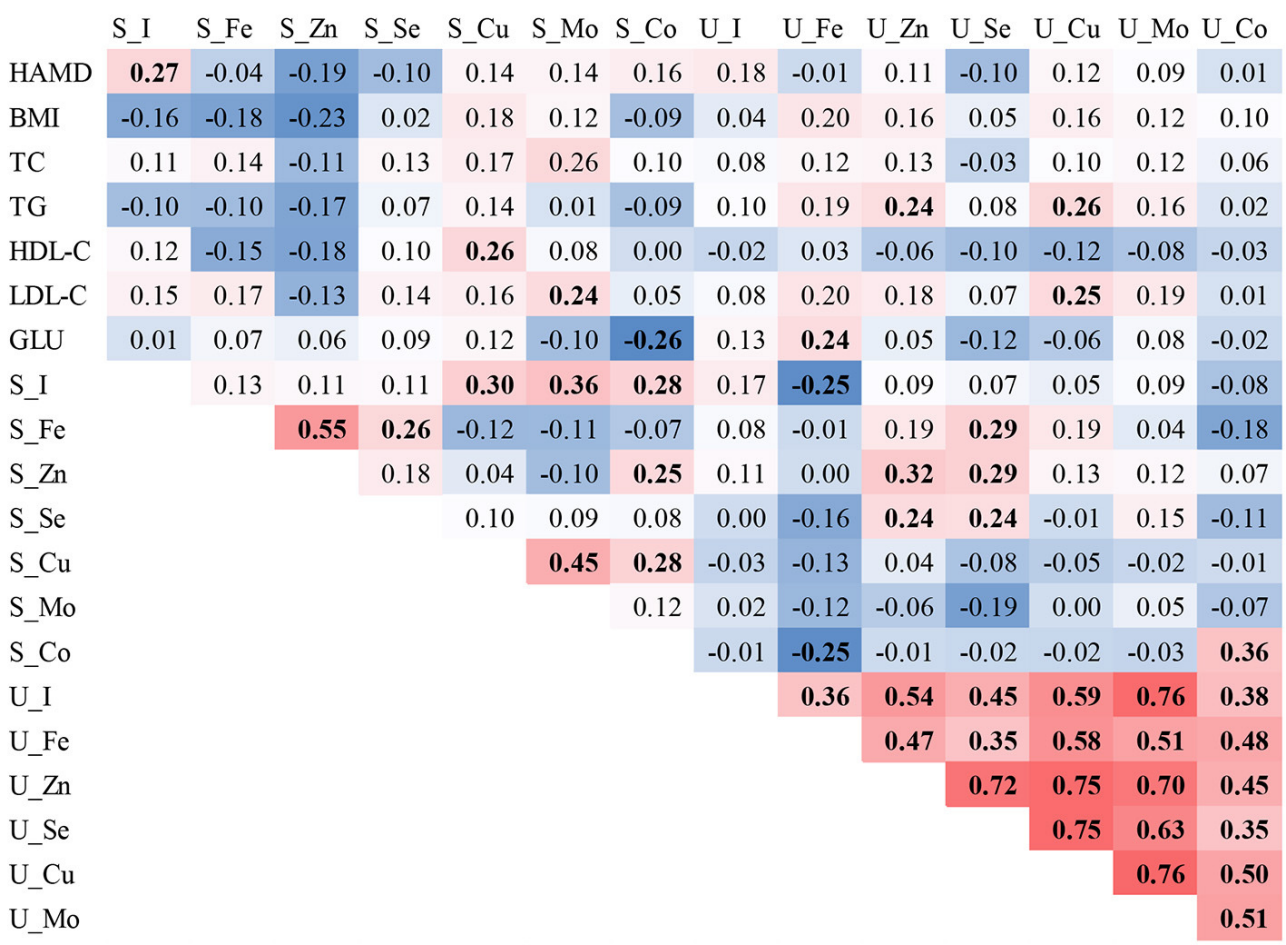
Spearman correlations of ETE levels in the serum and urine of individuals with MDD. The red background represents the positive correlations between the two compared variables, while the blue background represents the negative correlations. Bold font indicates statistical significance.

In the correlation analysis, we found that Cu in serum was positively correlated with high-density lipoprotein (*r* = 0.26), Mo with low-density lipoprotein (LDL-C) (*r* = 0.26), and Co with blood glucose (*r* = −0.26). In urine, Zn was positively correlated with total cholesterol (TG), Fe was positively correlated with blood glucose, and Cu was positively correlated with both TG and LDL-C. The ETEs in serum and urine were positively correlated with the HAMD-24 score (*r* = 0.27), except the element I in serum. Positive correlations were found among all ETEs in urine, with correlation coefficients ranging from 0.24 to 0.76, except for Fe, which was negatively correlated with I (*r* = −0.25) and Co (*r* = −0.25) in serum (see Figure 1).

## 4 Discussion

Essential trace elements are indispensable for the normal functioning of the human body. The correlation of many individual nutrients with mental depression have been confirmed (25), and the increased accumulation or lack of ETEs in serum will promote other metabolic pathways that can lead to many diseases and conditions of neurodevelopment (26). Serum and urine levels of seven trace elements were detected in a total of 147 individuals comprising MDD patients and HCs in the current study. The main finding of the study indicated that the level of I in serum was significantly lower in the MDD patients compared to the HCs; however, the level of I in urine was reversible, and it was hypothesized that I metabolism might be associated with the pathology of MDD. Additionally, Cu, Se, and Mo levels in serum were significantly lower in MDD patients compared to the HCs. In urine, Zn was significantly higher in the MDD patients compared to the HCs. Increasing levels of I, Cu, Se, and Mo in the serum might be protective against the development of MDD.

Iodine is one of the powerful antioxidants present in the body, and it is involved in the production of thyroid hormones (including thyroxine and triiodothyronine), which are essential for maintaining the body's metabolism and brain development (23). Globally, I deficiency is the most common cause of thyroid disorders (27), and patients with thyroid disorders more often report anxiety and depression compared to the general population (28). Only few studies reported the relationship between I intake and depression; however, the potential mechanisms were not well-clarified (19). We found that the concentration of I in the serum was significantly lower in MDD patients compared to HCs, while the opposite trend was observed in urine samples of our study. Similar to a previous study, our findings indicated that habitual low I intake is associated with an increased incidence of perinatal emotional distress and depression (19). Our study hypothesized that with major depressive disorder, decreased serum I uptake would result in increased urinary iodine levels.

Selenium is one of the most important essential trace elements of selenoproteins, which were found involved in the protective process against oxidative stress within the brain and the nervous system (29–31). It can cause depression-like behavior, neuroinflammation, and oxidative imbalance induced by acute restraint stress (15). The level of Se in serum was lower in MDD patients compared to the HCs, as found in our study. Our results are consistent with the previous findings that people with Se deficiency are more likely to suffer from depression (32). Furthermore, the findings suggest that low levels of Se in serum and urinary I concentrations in patients with nodular goiter may contribute to the development of anxiety and depression independent of thyroid function. In these patients, Se and I supplementation may help prevent anxiety and depression (33).

The key biochemical role of copper is to assist in the preservation of hematopoietic activity and to control energy metabolism and neurobehavioral and immune function (34). Excessive or insufficient amounts of Cu ions can lead to health problems, and individuals who have a large buildup of Cu ions in their bodies are known to suffer from Wilson's disease as well as from mental illness, anxiety, and depression (35). Consistent with other studies (36, 37), our current study finds that the concentration of Cu in MDD patients is lower than that in the HCs (38). On the other hand, some other studies reported that there was no significant difference in the serum concentration of Cu between the MDD patients and HCs (39–41). In this study, the Zn levels in serum were slightly higher in MDD patients compared to those in the HCs (although not statistically different). It has been shown that elevated Zn intake suppresses serum Cu levels (42).

Molybdenum is known to be essential for plants, animals, and humans (43). Mo has been recognized as an essential component of the enzymes, xanthine oxidase and sulfite oxidase (44). In contrast to our study, where the Mo levels in serum were lower in MCC patients compared to HCs, another study examining Mo and depression found no significant difference between the two groups. Additionally, this study observed that the concentration of Mo in urine was significantly higher in controls compared to that in depressed individuals (44, 45); however, no significant difference in urinary Mo levels between MDD patients and HCs was found in the current study.

Zinc is known to be an important trace element for many biochemical and physiological processes and promotes brain development, function, and cellular metabolism (46, 47). Studies have shown that low serum Zn levels in depressed patients may affect the ratio of neuronal biochemical metabolites in the patient's right prefrontal cortex and right tonsillar nucleus, leading to neuronal dysfunction, which may play a potential role in the pathophysiology of depression (22). Most of the current research studies conducted on Zn and depression has focused on serum or dietary intake, with fewer studies examining urinary Zn levels and depression. Our study found that the Zn level in urine was significantly higher in MDD patients compared to HCs. Furthermore, it is hypothesized that there is a link between the high loss of Zn in the body and the development of depression, although the exact pathology remains unclear. Moreover, previous evidence reported a decreased concentration of Zn may be a marker of traits in drug-resistant depression (48). Thus, drug-resistance should be considered for exploring the association between Zn and depression.

### 4.1 Limitations

The study has certain limitations that need to be considered. Although the study analyzed the levels of ETE in serum and urine, it was not possible to determine the relationship between the other trace elements such as Fe, Se, Mo, and Cobalt and depression. This is because the levels of ETE in other parts of the body such as nails and hair are also linked to mental disorders such as depression. The exclusion criteria did not include inflammatory diseases, which may significantly affect the levels of ETEs in serum and urine. As a cross-sectional study, it was challenging to establish a causal relationship. Therefore, some of the speculations in the study, such as the relationship between Cu and Zn and their effect on depression, should be considered with caution. Moreover, based on self-reported data, our participants' illness status was determined, and many residue cofounders might as well affect the findings of the study. Finally, to justify the findings of this study, larger samples are needed.

## 5 Conclusion

The levels of I in serum and urine were found to be reversed between MDD patients and healthy controls. In MDD patients, the levels of I in serum were lower compared to healthy controls. The study also found that the Cu concentration level in serum was significantly lower in MDD patients compared to HCs. In addition, significantly higher concentrations of Zn in urine may be linked to major depression. These findings imply the importance of essential trace elements in taking precautions against the major depressive disorder in adults. The levels of ETEs, especially I, Cu, and Zn, in the body, are crucial for preventing or alleviating depression and maintaining good health.

## Data availability statement

The raw data supporting the conclusions of this article will be made available by the authors, without undue reservation.

## Ethics statement

The studies involving humans were approved by Medical Ethics Committee of Zhumadian Second People's Hospital. The studies were conducted in accordance with the local legislation and institutional requirements. Written informed consent for participation in this study was provided by the participants' legal guardians/next of kin.

## Author contributions

JF: Conceptualization, Methodology, Writing – original draft. QW: Formal analysis, Methodology, Writing – original draft. NW: Methodology, Writing – original draft. SL: Project administration, Writing – review & editing. HZ: Software, Visualization, Writing – review & editing. YZ: Writing – review & editing. HG: Data curation, Writing – review & editing. FW: Writing – review & editing. LH: Data curation, Writing – review & editing. SX: Data curation, Writing – review & editing. BC: Conceptualization, Funding acquisition, Writing – review & editing.
