# Supplementary material for: Serum and urinary essential trace elements in association with major depressive disorders: a case–control study
Source: Front Psychiatry. 2023 Dec 1;14:1297411. doi: 10.3389/fpsyt.2023.1297411 (PMC10722235; doi:10.3389/fpsyt.2023.1297411)
Supplement: Supplementary file 1 [file Table_1.DOCX]

**Supplemental Table 1.** Instrument conditions for different instruments

| Equipment | Instrument conditions |
| --- | --- |
| 7700x | Carrier gas flow rate: 1.0 L/min, He gas flow rate: 4.5 ml/min, RF power: 1550W, Integration time: 300ms, Scanning mode: 3 Points |
| Elan DRC II | Atomizing gas flow rate: 1.05 L/min, auxiliary gas flow rate: 1.80L /min, plasma gas flow rate: 18.0 L /min, RF generator power: 1100w, residence time: 50-100ms, sample lifting volume: 1.1ml/min, scanning mode: single-point peak hopping, resolution: 0.7-0.9amu |
